# Raw diffraction data preservation and reuse: overview, update on practicalities and metadata requirements

**DOI:** 10.1107/S2052252516018315

**Published:** 2017-01-01

**Authors:** Loes M. J. Kroon-Batenburg, John R. Helliwell, Brian McMahon, Thomas C. Terwilliger

**Affiliations:** a Crystal and Structural Chemistry, Bijvoet Center for Biomolecular Research, Utrecht University, Padualaan 8, Utrecht, CH 3584, The Netherlands; b School of Chemistry, Faculty of Engineering and Physical Sciences, University of Manchester, Brunswick Street, Manchester M13 9PL, UK; c International Union of Crystallography, 5 Abbey Square, Chester CH1 2HU, UK; d Bioscience Division, Los Alamos National Laboratory, Mail Stop M888, Los Alamos, NM 87507, USA

**Keywords:** raw diffraction data, data archiving, metadata descriptors for raw data, diversity of crystallographic instrumentation

## Abstract

A topical review is presented of the rapidly developing interest in and storage options for the preservation and reuse of raw data within the scientific domain of the IUCr and its Commissions, each of which operates within a great diversity of instrumentation. A résumé is included of the case for raw diffraction data deposition. An overall context is set by highlighting the initiatives of science policy makers towards an ‘Open Science’ model within which crystallographers will increasingly work in the future; this will bring new funding opportunities but also new codes of procedure within open science frameworks. Skills education and training for crystallographers will need to be expanded. Overall, there are now the means and the organization for the preservation of raw crystallographic diffraction data *via* different types of archive, such as at universities, discipline-specific repositories (Integrated Resource for Reproducibility in Macromol­ecular Crystallography, Structural Biology Data Grid), general public data repositories (Zenodo, ResearchGate) and centralized neutron and X-ray facilities. Formulation of improved metadata descriptors for the raw data types of each of the IUCr Commissions is in progress; some detailed examples are provided. A number of specific case studies are presented, including an example research thread that provides complete open access to raw data.

## Abstract 2

There are rapid developments in storage options for the preservation and reuse of raw data within the scientific domain of the IUCr and its Commissions, each of which operates within a great diversity of instrumentation. Formulation of improved metadata descriptors for the raw data is under way. Science policy makers strive towards an ‘Open Science’ model within which crystallographers will increasingly work in the future. Case studies are described that show the community’s progress.

## 1. Introduction and overview   

### 1.1. Context   

Recent years have seen a growth in interest in retaining raw diffraction data sets collected for the determination of crystal and molecular structures. This interest has arisen spontaneously within the crystallographic community on a number of fronts. For example, raw data sets are valuable for developing new methods of structure determination and for benchmarking of software algorithms (Terwilliger & Bricogne, 2014 ▸); they are sometimes important for validating the interpretation of structural features; and increasingly they repay closer study, whether for allowing data analysis at higher resolution than used in the original work, understanding the presence of multiple lattices present in a crystal, or deducing details of correlated motions or disorder from the diffuse scattering that is largely ignored in determining Bragg peak positions and characteristics.

In parallel, the evolution of science policy in the wider world is prompting closer scrutiny of the whole practice of research data management, and there are a growing number of mandates to retain the raw data underpinning any experimental study and to make it available to other researchers. By early 2016, all UK scientific research councils had stated positions on data management, access and long-term curation (Digital Curation Centre, 2016 ▸; Research Councils UK, 2015 ▸). A useful summary of US Federal Funding Agency requirements for scientific data management is hosted by Northwestern University Library (2016 ▸). A noteworthy recent proposal calls for a European Open Science Cloud for Research (Jones, 2015 ▸).

Different communities have different ideas of what data they value most – and, indeed, of what constitutes ‘data’. The USA’s National Science Foundation (NSF) makes this explicit in its published ‘Frequently Asked Questions’ (National Science Foundation, 2010 ▸):

*1. What constitutes ‘data’ covered by a Data Management Plan?*

*What constitutes such data will be determined by the community of interest through the process of peer review and program management. This may include, but is not limited to: data, publications, samples, physical collections, software and models.*

In consequence, there is great variety amongst different scientific disciplines in their approaches to data management and retention, and therefore in the availability of public repositories and in the software tools to manage deposition, access and reuse. Nevertheless, two themes recur in the various published mandates and best-practice guidelines: the importance of persistent identifiers for data sets, and the vital need to characterize them as fully as possible by appropriate metadata.

Crystallography is generally regarded as a science that has its house in good order regarding data management, validation, access and reuse. This is largely true so far as ‘derived’ data (by which we mean atomic positional coordinates and displacement parameters resulting from structure determinations) and associated publications are concerned. It is more debatable where processed diffraction data are concerned – the post-experiment processed data (typically structure factors) that form the basis of the atomic and molecular structure determination and subsequent refinement leading to a structural model. Some journals require deposition of structure factors in support of any publication, and the Protein Data Bank (PDB; Berman *et al.*, 2000 ▸) requires structure factors to be deposited along with the atomic coordinates. However, these are usually the final set of structure factors used in refinement, and may lack information discarded when merging symmetry-related diffraction peaks, or excluded for other reasons from early cycles of refinement. The PDB *will* accept unmerged processed intensity data, and there are community recommendations encouraging their deposition (International Structural Genomics Organization, 2001 ▸), but the practice is not yet universal in macromolecular crystallography. For small-unit-cell crystal structures, even journals that accept structure factors have not hitherto required unmerged intensities. However, there is growing recognition that they are important, both for further development of the *checkCIF* validation carried out during the peer review process, and indeed to encourage future researchers to revisit and re-evaluate the published results, perhaps when new ideas or tools become available (A. Linden, personal communication).

However, historically there has not been a tradition of retaining the raw X-ray diffraction images collected by electronic detectors, although centralized neutron facilities have long-standing traditions of raw data preservation. In recent years the practices nurtured by the neutron facilities have been spreading; each type of large-scale centralized instrumental facility (synchrotrons and latterly free-electron lasers, as well as neutron reactors) has begun to move towards raw data preservation. This trend has been encouraged by rapidly improving electronic data-handling procedures.

In 2011, the International Union of Crystallography (IUCr) established a Working Group to explore the merits and challenges of retaining the initial experimental data. This group, the Diffraction Data Deposition Working Group (DDDWG), has conducted a number of consultations, discussion meetings and workshops to explore the topic. A set of papers published in *Acta Crystallographica Section D* (Terwilliger, 2014 ▸) provided an overview of the reasons for archiving raw data in the field of macromolecular crystallography, models for doing so on a routine or large-scale basis, current practical initiatives, and the potential benefits for improving macromolecular structure models.

These papers also highlighted the importance of assigning persistent identifiers to data sets to facilitate their management and long-term curation, and to ensure that each data set was characterized by rich metadata, both to facilitate discovery and to allow effective scientific reuse (Guss & McMahon, 2014 ▸; Kroon-Batenburg & Helliwell, 2014 ▸).

In the remainder of this *Introduction*, we introduce a recent workshop that concentrated on metadata in crystallographic and related experiments; we review the arguments for depositing raw data as a routine practice; and we place these activities in the context of global science policy initiatives. The paper then looks in more detail at the current and evolving mechanisms for the deposition of raw experimental data (especially X-ray diffraction images); at detailed requirements for metadata that describe archived data sets, in order to ensure the reproducibility of the derived scientific results; and at the next steps forward.

### 1.2. Improving the metadata   

To focus on the metadata issues, the DDDWG conducted a two-day workshop at Rovinj, Croatia, in August 2015. A complete record of the workshop is maintained online at http://www.iucr.org/resources/data/dddwg/rovinj-workshop and a number of articles arising from the meeting are in preparation. We detail here some specific outcomes from the workshop.

#### 1.2.1. Efforts of the IUCr Commissions   

**Figure 1:**
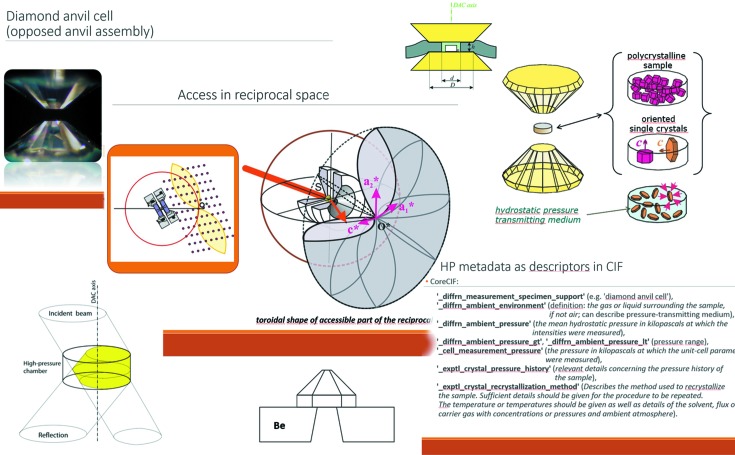
Montage of slides from Kamil Dziubek’s presentation at the Rovinj workshop, illustrating aspects of diffraction experiments under high pressure and other non-ambient conditions that need to be well characterized and recorded. (Graphics courtesy of Ronald Miletich-Pawliczek, University of Vienna.)

The IUCr manages its scientific mission through a number of Commissions, each responsible for a particular topic area within crystallography. The DDDWG has requested each Commission to consider its own needs for defining metadata for raw experimental data within its field. Among those that have been most active in responding to this request are the Commission on XAFS (Ravel *et al.*, 2012 ▸); the Commission on Small-Angle Scattering (Jacques *et al.*, 2012 ▸); the Commission on High Pressure (Fig. 1 ▸); and the Commission on Biological Macromolecules (*e.g.* Gutmanas *et al.*, 2013 ▸).

The International Centre for Diffraction Data (ICDD, Pennsylvania, USA; http://www.icdd.com) has been active in the harnessing of raw powder diffraction data sets for some time and reported to us at ECM29 in Rovinj (August 2015) that they have now incorporated over 10 000 raw powder diffraction data sets into the Powder Diffraction File. They note that one-dimensional data sets are generally reasonably well characterized in terms of the experimental metadata catalogued in the powder CIF (pdCIF) dictionary (Toby, 2005 ▸), but that interpretation of two-dimensional diffraction images is hampered by a lack of consistency in reporting such characteristics as goniometer axes, detector dark current, distortion and other corrections (T. Fawcett, personal communication; see also Section 1.2.2). The Commission on Powder Diffraction is planning further work on neutron powder diffraction raw data and will liaise with the Commission on Neutron Scattering as appropriate. The Commission on Structural Chemistry has had enthusiastic participants in events convened by the DDDWG in Madrid, Bergen and Rovinj.

#### 1.2.2. Characterizing X-ray diffraction images   

The class of experimental data sets that most closely fits the original remit of the DDDWG is X-ray diffraction images collected from CCD or pixel detectors. A good catalogue of the metadata needed, in general, to interpret a raw image data file was given by Kroon-Batenburg & Helliwell (2014 ▸). Many of the individual items required are defined in the imgCIF dictionary (Bernstein, 2005 ▸), and there have been partial implementations of some of them in so-called ‘mini-CBF’ headers of image files written by a number of commercial detector systems. However, this has not been done in a consistent way between vendors nor even across the entire product range of individual vendors. (CBF, the crystallographic binary file, and imgCIF, its pure ASCII counterpart, are equivalent implementations of the CIF ontology for diffraction images.)

Increasingly, images are being stored using the HDF5/NeXus data format (Könnecke *et al.*, 2015 ▸), and although the physical format of the data file should not affect its ability to store specific structured information (Hester, 2016 ▸), some effort will be needed to ensure that the CIF and NeXus data representations are equally capable of storing the appropriate experimental metadata. Significant effort to achieve this at the technical level has already been invested following participation in an earlier workshop by representatives of COMCIFS (Committee for the Maintenance of the CIF Standard) and NIAC (NeXus International Advisory Committee), the bodies responsible for managing the CIF and NeXus data formats, respectively (Bernstein *et al.*, 2013 ▸). Nevertheless, presentations at the Rovinj Workshop by Kroon-Batenburg (https://youtu.be/XXFDlNn21SY) and by Minor (https://youtu.be/eQbs9sB_pOM) emphasized that there is still a long way to go before the myriad different formats generated by commercial electronic position-sensitive detectors do contain the necessary common metadata to allow for easy interpretation and management (see further discussion in Section 3.2).

The arrival of the new Dectris Eiger pixel detector, with its colossal increase in diffraction image data rates, has highlighted the importance of efficient data format and metadata recording, not only for diffraction data processing on a synchrotron or X-ray laser beamline, but also for subsequent processing outside the facility, and ultimately for reprocessing/reanalysis from a raw data archive as may be needed. The various issues have been highlighted in detail in a discussion thread on the CCP4bb mailing list in early March 2016 (involving, amongst others, G. Winter, A. Förster, H. J. Bernstein, C. Vonrhein and G. Bricogne).

### 1.3. The case for raw data deposition   

We summarize the case for routine storage and retrieval of raw data to emphasize its potential value to the community. At the same time we acknowledge the cost and other practical constraints of storing all collected data sets indefinitely, and we are unable to give a definitive indication of where the balance might lie between archiving and discarding raw data. However, we show in Section 1.4 that there are discernible trends towards storing more data sets than we might have expected in the early work of the DDDWG.

There is a broad philosophical view of the importance of access to raw diffraction data, namely that science requires the ability to conduct a comprehensive analysis through one’s own eyes and not the lens of someone else. Raw diffraction images offer several opportunities for improved or novel science. They permit the analysis of data at higher resolution than used in the original work [allowing comparisons not only among data processing software (Tanley *et al.*, 2013 ▸), but also in the effectiveness of structure determination and refinement with ever weaker data beyond normal limits]. Raw data sets can serve as benchmarks in developing improved methods of analysis. They allow checking of the interpretation of the symmetries of the crystals, and detailed analysis of diffraction from multiple lattices present in the crystals. More generally, they promote the study of the diffuse scattering that reflects correlated motions or disorder of atoms in the crystals, namely the ‘structural dynamics’.

The retention of raw data can be seen as complementing the extensive archives of derived data (*i.e.* cell parameters, molecular coordinates, anisotropic displacement parameters) and processed data (structure factors, Rietveld refinement profiles) in the crystallographic databases. The contributions of the former are very well understood: they form part of the scientific record, they lead to database-driven discovery, *e.g.* in understanding protein–ligand interactions, they lead to new pathways to synthesis, improvements in manufacturing and better understanding of energetics, and they have use in identification and indexing applications (*e.g.* in forensic science).

Until the advent of CIF and the automated structure validation checks with the *checkCIF* suite (Strickland *et al.*, 2005 ▸) that it enabled, many structures were published which required subsequent correction. Often, the interpretation of the results produced molecular structures that were broadly correct, but overlooked higher lattice symmetries. Such examples were best detected and corrected through access to the deposited structure factors (well illustrated by Marsh *et al.*, 2002 ▸).

So, broadly speaking, structure validation (the credibility of a structural model, both in its adherence to norms of geometric configuration and its derivation from X-ray diffraction images) can be carried out with reference to the derived data sets (the structural coordinates) and the structure factors alone, and this has been the practice in various crystallography journals for a considerable length of time. However, the availability of the raw data (*i.e.* original diffraction images) can enhance structure validation in the following ways:

(i) The structure can be re-refined, perhaps making use of diffraction peaks that were excluded because the processed diffraction data were truncated at an arbitrary resolution limit. Retention of the original data also permits re-evaluation of the space-group symmetry, which is normally settled upon during an early stage of conventional refinement.

(ii) Data reduction is often performed according to established protocols, but retention of the original images allows the opportunity to test those protocols, especially if there is any suspicion of systematic bias. Indeed, statistical analysis of a collection of stored raw images may allow the detection of systematic biases that are not at all apparent in individual experiments. Further, the availability of large collections of raw data sets allows periodic recalibration of solution methods and the development of new methods to tackle data sets that have previously been resistant to conventional solution.

(iii) Attention to diffuse scattering between the diffraction spots allows insight into correlated motions or disorder of atoms in crystals. This might involve quasicrystalline behaviour, determination of incommensurate modulation or multi-phase representation, macromolecular motions or conformational changes *etc*.

Note that these benefits may not be apparent for every structure, and the cost–benefit calculus informing policies of routine deposition has still to be determined by the community and funding bodies (Guss & McMahon, 2014 ▸). It may be that there are different entry points where the potential benefits can be most readily realised, *e.g.* by making available the experimental data for ‘difficult structures’ that have proved impossible to refine satisfactorily.

However, more-or-less routine deposition of primary data would help to improve the quality and reliability of the scientific record (Minor *et al.*, 2016 ▸). It would allow closer scrutiny of scientific deductions by peer reviewers prior to publication; it would allow for revisiting and revising structural models already in the databases, as new techniques are developed – *e.g.* the notion of ‘continuous improvement of macromolecular structure models’ (Terwilliger, 2012 ▸); it allows reanalysis of a structure or series of structures independent of an author’s interpretational bias (B. D. Bax, personal communication); and it provides the experimental evidence needed to support any claims made by the publishing author. In this last role, it helps to guard against the use of the wrong data set, either through error or deliberate intention.

### 1.4. Deposition imperatives and opportunities   

**Figure 2:**
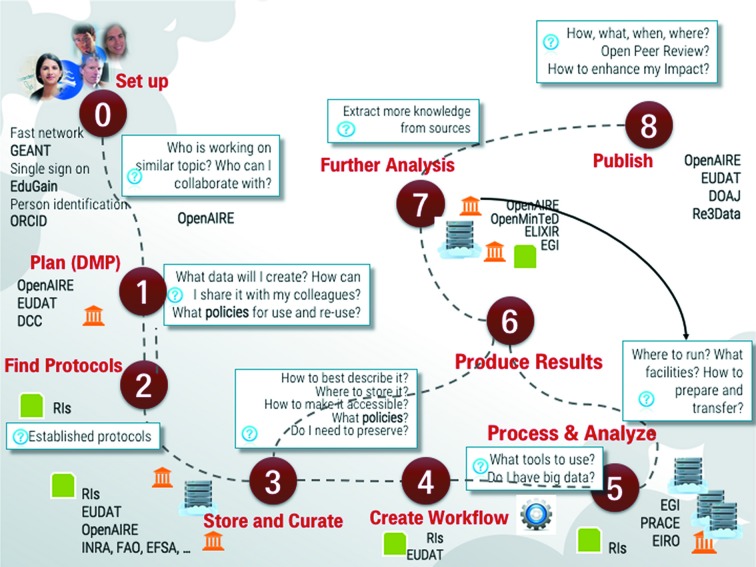
A graphic linking data publishing and management workflow to EU research infrastructural components. Part of a presentation introducing the European Open Science Cloud for Research (illustration courtesy of Natalia Manova for the European OpenAIRE project).

As previously mentioned, there have been developments since the DDDWG was established in the climate for data deposition and sharing, both in the wider scientific world and in the field of crystallography and related structural sciences. The benefits of open data (*i.e.* collecting research data arising from publicly funded scientific research and making it available for reuse without charge to the end user) have been reiterated in recent years in international, governmental and scientific policy discussions and practical initiatives. Among a few portal web sites of note are the United Nations data portal (UNdata: http://data.un.org), the US Government open data site (https://www.data.gov) and the federated ‘Global Science Gateway’ http://worldwidescience.org. Calls for implementation include ‘The Good Growth Plan’, a collaboration for agricultural development involving the UK Open Data Institute (ODI; https://theodi.org) and Syngenta; the European Open Science Cloud (EOSC), a European Union strategy for linking research networks, data storage facilities and computing resources across the continent (Jones, 2015 ▸; Fig. 2 ▸); and an Open Data Accord (Science International, 2015 ▸) launched by the International Council for Science (ICSU), the InterAcademy Partnership (IAP), The World Academy of Sciences (TWAS) and the International Social Science Council (ISSC).

Although these various initiatives are very diverse in their objectives, collectively they are raising the perceived importance of data repositories to research funders, to researchers who are encouraged or in some cases mandated to deposit their data in robust and durable repositories, and to other researchers who are becoming increasingly aware of the availability of other data sets and their potential usefulness to their own work. A gradual change in cultural attitudes to research data is taking place.

Since the DDDWG was established in 2011, there have been a number of developments, some catalysed by these high-level initiatives, that have increased the options for deposition of diffraction images:

(i) The number and scope of university data repositories has expanded.

(ii) The European Synchrotron Radiation Facility (ESRF; Grenoble, France) has launched a Data Archive, in which every raw data set measured can be associated with a registered DOI.

(iii) The Zenodo science data archive, hosted on the extremely high capacity CERN storage system, has gathered momentum.

(iv) A repository for diffraction experiments used to determine protein structures has been established as part of the US National Institute of Health’s BD2K (Big Data to Knowledge) programme (Grabowski *et al.*, 2016 ▸); it is run by Wladek Minor’s group at the University of Virginia, USA (http://www.proteindiffraction.org/).

**Figure 3:**
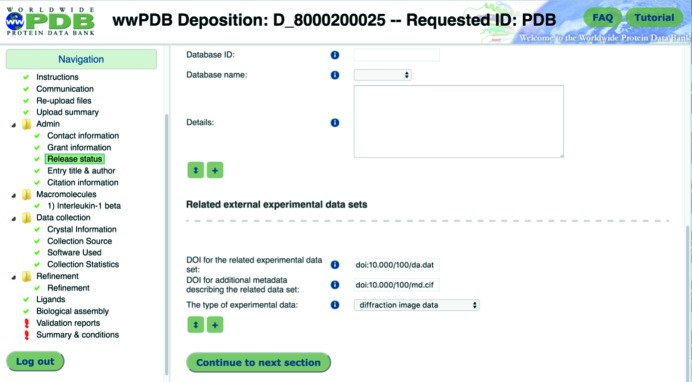
Online form allowing PDB depositors to link experimental data sets and their associated metadata with a deposited macromolecular structure.

(v) The Structural Biology Data Grid (SBDG) has been established as a diffraction data publication and dissemination system for structural biology (Meyer *et al.*, 2016 ▸).

(vi) The Protein Data Bank (PDB) now requests the DOI (digital object identifier) for raw data and metadata for raw data during a deposition (Fig. 3 ▸).

(vii) *IUCrData* (an IUCr data service, initially handling derived data sets) has been launched.

Some of these are described in more detail in Section 2.2.

## 2. Mechanisms for raw diffraction data preservation   

We review some of the *de facto* repositories that are currently hosting, and in many cases providing access to, experimental data sets in our domain.

### 2.1. Institutional data repositories. Case study: University of Manchester   

**Figure 4:**
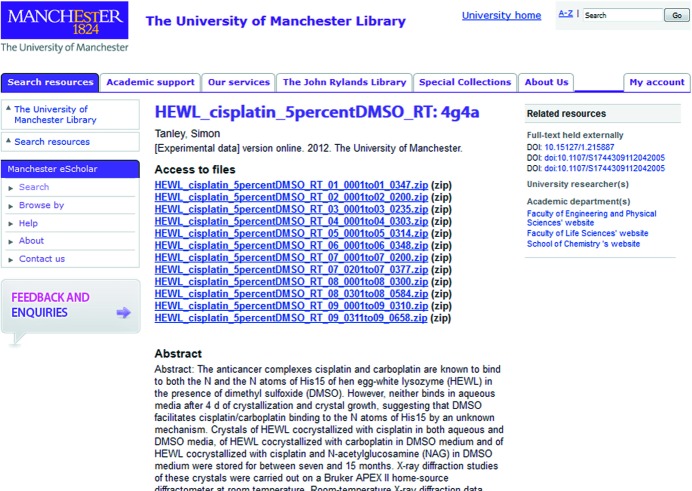
Manchester University Library access record for experimental data sets associated with a published research article. Links are provided to the published article in the ‘Related resources’ column.

**Figure 5:**
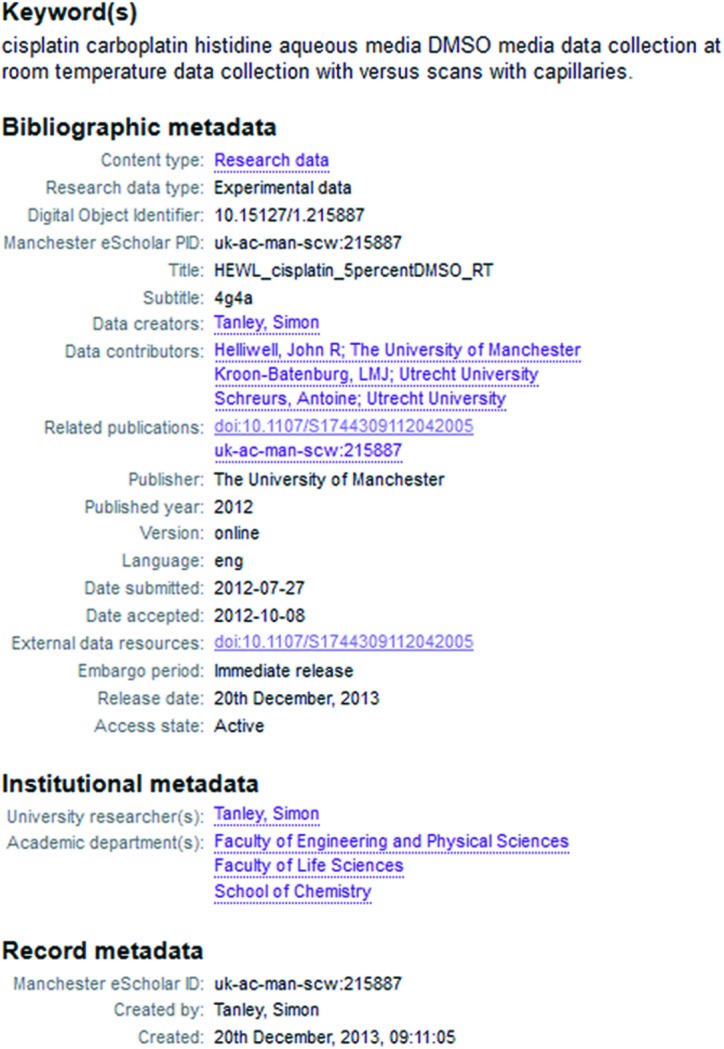
Classification-level metadata associated with experimental data sets archived at the University of Manchester Data Library. These identify the archived data sets and provide links to related resources.

The meticulous approach of the University of Manchester makes one of us (JRH) feel very fortunate to be working in this research environment. In researching the binding of the anti-cancer agent cisplatin to histidine [which has received intense interest; see, for example, Messori & Merlino (2016 ▸)], JRH’s research group made the raw diffraction data open access at the University of Manchester institutional data repository. Fig. 4 ▸ illustrates the data access record within the Library system, while Fig. 5 ▸ illustrates the classification-level metadata required by such a repository. This type of institutional cataloguing and archive is increasingly characteristic of modern data archive initiatives. In addition, we have followed the standard community data deposition requirements of depositing coordinates and processed diffraction data at the Protein Data Bank. To permit the widest possible access to our work, we have also been able, *via* the EPSRC funding we have had, to publish the bulk of our articles reporting our results as ‘gold’ open access (*i.e.* the full peer-reviewed articles of record can be accessed without a journal subscription) in *Acta Crystallographica Sections D* and *F*.

In becoming pioneers of making both our raw diffraction data and our data and model interpretations fully open (Table 1 ▸), thus achieving a rare breadth and depth of openness within a focused research theme, our research has received a gratifying amount of detailed interest. There have been many downloads of these raw data, both from their original web location at Utrecht University and subsequently from the University of Manchester. The download totals for each year from Utrecht were: 2012 17 GB, 2013 47 GB, 2014 57.69 GB and 2015 31.47 GB; equivalent download information is not available from the University of Manchester. One such raw data download featured in a new publication (Shabalin *et al.*, 2015 ▸), a wide-ranging critique of the whole field of cisplatin binding to various proteins. This article suggested improvements to three of our cisplatin–lysozyme models in the PDB *via* three of their own alternative interpretations; two of these involved use of our processed diffraction data held at the PDB (4xan and 4mwk) and one of our raw data (4g4a in Table 1 ▸ and Fig. 4 ▸). We have accepted some of their recommendations and rejected others (Tanley *et al.*, 2016 ▸). Some of these points of ‘data debate’ also suggest a lack of mature community standards, even within one journal (Tanley *et al.*, 2015 ▸), but they also show a way forward for discussions to be conducted, *e.g.* within IUCr journals. In other aspects, it shows the benefits of the continuing pursuit of improved methods of analysis and a better understanding of the role of weak data in improving protein model refinements (Diederichs & Karplus, 2013 ▸), which we harnessed in detail in Tanley *et al.* (2016 ▸). Such improvements have arisen even in just the last few years, and illustrate the ‘young age’ of macromolecular crystallography, a field that is still clearly maturing as a technique.

### 2.2. General data repositories for structural biology   

The importance of data capture and archiving has been widely recognized around the world and several repositories are now available where nearly any researcher can, or will soon be able to, deposit their raw data and associated metadata for anyone in the world to view and download, subject of course to the natural constraints of file size and network bandwidth.

Two major publicly funded repositories are the Integrated Resource for Reproducibility in Macromolecular Crystallography (http://www.proteindiffraction.org) and the Zenodo repository (https://zenodo.org) for general scientific data. The former has been developed by the Minor group at the University of Virginia (http://olenka.med.virginia.edu/CrystUVa) and is supported by the US National Institutes of Health Big Data to Knowledge Initiative (https://datascience.nih.gov/bd2k). Zenodo has been developed by CERN (http://www.cern.ch) as part of the European Union OpenAIREplus initiative (http://www.openaire.eu).

Two additional private repositories are available for general use. The Harvard-based SBGrid organization (https://sbgrid.org) has developed a Structural Biology Data Grid (https://data.sbgrid.org) that can be used by any member of SBGrid to archive raw data and metadata. The ResearchGate scientific networking site (https://www.researchgate.net) allows researchers to share data (https://www.researchgate.net/blog/post/present-all-your-research-in-a-click).

#### 2.2.1. The Integrated Resource for Reproducibility in Macromolecular Crystallography   

The Integrated Resource for Reproducibility in Macromolecular Crystallography (Grabowski *et al.*, 2016 ▸) is a protein diffraction database that addresses the need for archival of crystallographic raw images, as outlined in the discussion above and in the *Acta Cryst. D* group of articles published recently (Terwilliger, 2014 ▸). This database currently includes over 2900 raw crystallographic data sets and associated metadata. Most of these are linked with a deposit in the Protein Data Bank (http://www.pdb.org; Berman, 2000 ▸) and many of them represent work from structural genomics projects (http://csgid.org, http://ssgcid.org, http://www.jcsg.org, http://mcsg.anl.gov, http://thesgc.org). The database is highly structured, with crystallographic metadata associated with each data set. A very useful feature of this service is that the web interface to the database shows a representative diffraction image from each data set, allowing a researcher to note quickly the characteristics of the diffraction from the crystals used in each data set, for example the order in the diffraction pattern, the presence of diffuse scattering and the extent of anisotropy in the diffraction pattern. The database can be searched based on PDB ID, resolution of diffraction, the location where data were collected, authors, and many other characteristics. It is planned for the database to be available for deposits and downloads by anyone. Every entry in the database has an assigned DOI that can be used to refer to the data and which provides a stable permanent link to the data, and the data deposited are not limited in file size. The metadata associated with the raw data are an integral part of the database, so that it may be practicable in the future to reprocess automatically much of the raw data in the database as new algorithms for data analysis become available (*cf.* Terwilliger & Bricogne, 2014 ▸).

#### 2.2.2. Zenodo   

The Zenodo archive is a general scientific archive developed by researchers at CERN as part of a European Union Framework 7 initiative. It provides a repository for scientific data sets in any field and has the unique feature that, as part of CERN, it has access to exceptional capacity for data storage and archiving. Though it is supported by the EU, researchers from anywhere in the world can archive their data and anyone can access the data. The Zenodo archive is designed to provide a resource for the many small scientific projects in the world that do not have an easy way to make their data available to the scientific community and, unlike the other databases discussed here, plans to charge a fee for larger-scale users. The archive currently has over 2500 data sets from all fields of science. Data sets can have multiple files, normally up to a total size limit of 50 GB; individual files can be a maximum of 2 GB in size. Each data set is assigned a DOI for permanent archiving and discovery, and is linked with metadata provided by the researcher.

#### 2.2.3. Structural Biology Data Grid   

The SBGrid organization provides access for researchers at many structural biology laboratories around the world to a packaged set of software that can be used in many areas of structural biology, including X-ray crystallography, cryo-electron microscopy, electron diffraction, small-angle scattering and other areas. SBGrid also provides access to cloud-based computing resources that carry out structural biology calculations. The Structural Biology Data Grid is a service recently started by SBGrid that allows any SBGrid researcher to archive raw data from any of the SBGrid structural biology areas. This database currently has over 240 data sets from 62 different institutions. The data can be viewed by anyone and crystallographic data sets can be downloaded by anyone, with cut-and-paste scripts for easy downloading of individual data sets. Each data entry has a unique DOI assigned, there are no limitations on file sizes, and metadata describing how to analyse the data are provided.

#### 2.2.4. ResearchGate   

ResearchGate is a commercial scientific social networking service that provides a simple mechanism for researchers to post their scientific papers and information about themselves, and for researchers to communicate about and discuss scientific topics. ResearchGate additionally allows researchers to archive scientific data sets for anyone to download. The data sets are assigned a DOI, and the size of individual files is limited.

### 2.3. Synchrotron, neutron and X-ray laser facility options   

There are now several striking examples of current and evolving practice in data capture and management across a range of large-scale facilities accommodating a variety of techniques and sciences. Among those we are aware of are the Australian Synchrotron (Clayton, Victoria, Australia), the ESRF, the Institut Laue–Langevin (ILL, Grenoble, France), the Diamond Light Source (Didcot, UK) and the ISIS neutron source at the Rutherford Appleton Laboratory (Didcot, UK). The Australian Synchrotron has led the world’s synchrotrons on data archival with its Store.Synchrotron data storage service for macromolecular crystallography (Meyer *et al.*, 2014 ▸). As well as diffraction image data archiving, it also supports users in their publications with linking to raw data sets *via* DOI registrations and, finally, the release of data sets for public analysis – something that, in the neutron community, the ILL is doing as well. There are also fine examples like Diamond that has so far retained all of its measured data. The ESRF has published a summary of its views on the era of Big Data at synchrotron radiation facilities in general and the challenges that today face the ESRF itself (ESRF, 2013 ▸). In an encouraging recent statement, it has announced a proactive data archiving policy (Andy Götz and colleagues from ESRF, personal communication).

There are still very significant challenges of data management in home laboratories and for medium-scale service providers such as the UK National Crystallography Service (Southampton, UK). In all these places, all the data from an experiment must be handled in the context of resource management, provenance, validation and bulk storage, all of which require ever greater volumes of metadata that should conform to widely accepted standards.

### 2.4. The data deluge   

One caveat that we apply to our encouraging survey of repository solutions is that, as technology advances, so the volume of data collected is increasing at a dramatic rate. Hence, while the entire download total from Utrecht University in 2015 was 31 GB, a single data set produced by an Eiger 16M detector currently operating on a synchrotron beamline could be over 70 GB. This suggests that centralized experimental facilities, with their large data storage capacities and gigabit internal networks, will continue to play an important role as first-choice repositories for quasi-routine retention of data sets. However, it may also become necessary to apply principles of ‘triage’, either at the point of data collection or in subsequent long-term storage allocation. Such triage might either delete certain data sets or retain some subset, according to a variety of possible criteria. An initial suggestion for a set of such criteria was proposed in the DDDWG online forum in 2011 (http://forums.iucr.org/viewtopic.php?f=21&t=57) but has yet to be developed by the community.

## 3. Metadata for raw data requirements   

### 3.1. A holistic metadata framework for crystallography   

Crystallography and related structural sciences are fortunate in having a standardized approach to data characterization and management, known as the Crystallographic Information Framework (CIF; Hall & McMahon, 1995 ▸). This has two components: a standard file format and data model (Hall *et al.*, 1991 ▸; Bernstein *et al.*, 2016 ▸), which facilitate data exchange between software programs, structural databases and publishing systems; and a set of ‘dictionaries’ that control the meaning of the tags associated with data values, and which can impose restrictions on data types and values where appropriate. These dictionaries collectively constitute the controlled vocabulary and associated definitions that represent the semantic meaning of a data file or stream – what is fashionably called the ‘ontology’ of a particular scientific domain.

Each CIF dictionary contains definitions relevant to a particular field or topic area, such as small-unit-cell structures determined by single-crystal diffractometry (the so-called ‘core’ dictionary), powder diffraction, biological macromolecular structures, modulated incommensurate structures, multipole electron density or diffraction images (Hall & McMahon, 2016 ▸). These compilations by topic take a comprehensive view of what may be termed ‘data’. Thus, the core dictionary contains items as diverse as a single atomic positional coordinate, the ambient temperature at the time the experiment was conducted, the convergence metrics of the least-squares refinement, the software used for generating molecular graphics, or the entire text of an associated scientific publication. That is, there is no differentiation between items that might normally be categorized as ‘raw’, ‘processed’ or ‘derived’ data, or that might be characterized as ‘metadata’.

**Figure 6:**
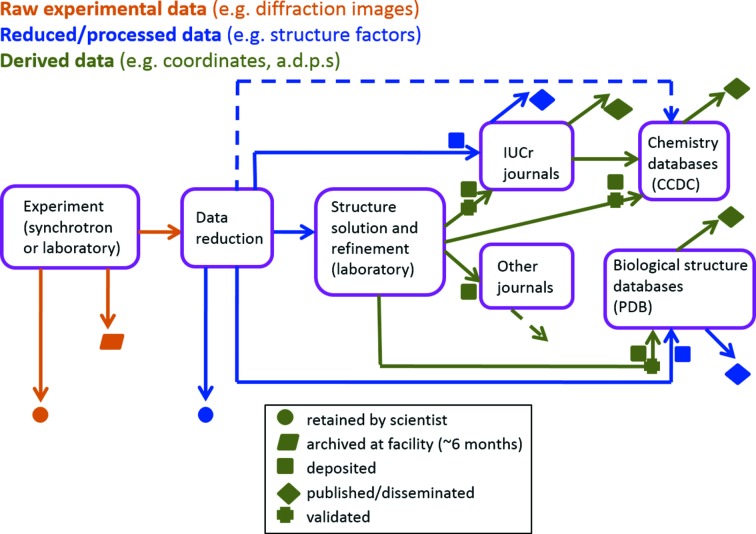
A coherent information flow in crystallography. CIF ontologies characterize data at every stage of the information processing life cycle, from experimental apparatus to published paper and curated database deposit.

The advantage of this lack of differentiation is that *all* the information needed to interpret, validate or reuse a data set can be stored in a single file; and this can make it easier to collect and verify such information during the course of an experimental workflow. Fig. 6 ▸ illustrates how the CIF ontologies inform the ‘coherent information flow’ at every stage of the information processing lifecycle in a typical structure determination experiment. In practice, not all real-world workflows use CIF as their actual mechanism for capturing data and metadata. For example, in large instrumental facilities, information about a particular experiment might be collected within a unified content management system developed by the facility to accommodate a wide range of different scientific experiments (Matthews *et al.*, 2010 ▸). Similarly, to manage the high-throughput data acquisition requirements of modern detectors, images may be generated as binary HDF5 files, or in proprietary formats.

Nevertheless, all raw data sets and associated metadata can, in principle, be converted into CIF representations, which might be a practical benefit for archiving purposes (*i.e.* to use a single standard representation), or at the very least can demonstrate what important metadata are missing, by comparison with the comprehensive CIF dictionary compendia of what can and should be collected.

Various IUCr Commissions are continuing to compile metadata definitions relevant to their field of interest in the form of CIF dictionaries. In addition to those listed by Hall & McMahon (2016 ▸), a small-angle scattering dictionary (sasCIF) has recently been published (Kachala *et al.*, 2016 ▸); work is well advanced by the IUCr Commission on Magnetic Structures to characterize magnetic structures and their underlying symmetries (magCIF); and the Commission on High Pressure has an active working group defining essential aspects of the experimental setup needed in non-ambient crystallography.

As mentioned before, the imgCIF dictionary describes an actual format for storing raw diffraction data. However, it also includes a rather complete set of data items that, if fully populated and used in conjunction with other items in the core or macromolecular CIF dictionaries, can fully describe the experimental apparatus and operating parameters, thus permitting a complete interpretation of archived images in this format. The imgCIF format itself is relatively little used, largely because of the speed requirements in modern detectors which require different data acquisition strategies. However, there is an ongoing effort to define metadata terms in the increasingly common NeXus format (Könnecke *et al.*, 2015 ▸) that are in concordance with the experimental metadata items defined in the imgCIF dictionary.

### 3.2. The diversity of instrumentation   

In this section we examine the specifics of some of the problems encountered in practice with missing or poorly characterized metadata. The availability of metadata in image headers and their interpretation by software developers has been discussed previously (Tanley, Schreurs *et al.*, 2013 ▸; Kroon-Batenburg & Helliwell, 2014 ▸). It can safely be concluded that metadata information is often lacking or is ambiguous, *i.e.* can be interpreted in different ways. Hardware manufacturers may use different words for the same physical parameter or its units, and it is all in the hands of the software developers to make correct use of the metadata information and fill in the missing parts, simply by acquired knowledge or by trial and error. We refer to the supporting information in the paper by Kroon-Batenburg & Helliwell (2014 ▸) for a discussion between Kay Diederichs, Toine Schreurs and Loes Kroon-Batenburg about φ scans around an axis not perpendicular to the X-ray beam on a fixed χ goniometer. Though sufficient information was available in the header, the *XDS* software (Kabsch, 2010 ▸) ignored most of it and used knowledge of the (usual) instrumental set-up, which in this case did not suffice. Initially the raw data, which are now on the Manchester University Library archive, were stored on a website at Utrecht University (http://rawdata.chem.uu.nl) and we added a photograph of the experimental set-up as metadata to resolve the ambiguity of the goniometer, *e.g.* is the spindle axis pointing up or down?

We should distinguish between diffraction equipment designed to be used in combination with the manufacturer’s software, which adequately handles metadata information, and assembled instruments like those on a synchrotron beamline. In the first case, taking the data to another place for use with third party software may give rise to problems, as described by Tanley, Diederichs *et al.* (2013 ▸). The image headers at best contain the type of goniometer (*e.g.* ‘MACH3 with KAPPA’ for Bruker Proteum) but rarely are the orientations and dependencies of the four axes given. In the second case, commercial detectors (*e.g.* the Pilatus from Dectris) are installed on a beamline and it is the beamline control software, in close interaction with the detector software, that is responsible for writing information in the image headers. In this mixed environment not all metadata are captured. Usually, but not always, the wavelength, detector-to-sample distance, pixel size and number of pixels in either direction, rotation start angle and increment, and exposure time are given.

The most common problems with metadata, however, are related to the orientations of the goniometer axes and rotation directions, and the definition of the faster and slower directions in pixel coordinates with respect to the laboratory axes and the origin of the pixel coordinates; especially disturbing is the absence of or an incorrect beam centre (see below). Table 2 ▸ gives the goniometer definitions known to the *EVAL* software (Schreurs *et al.*, 2010 ▸) and shows their large variety.

**Figure 7:**
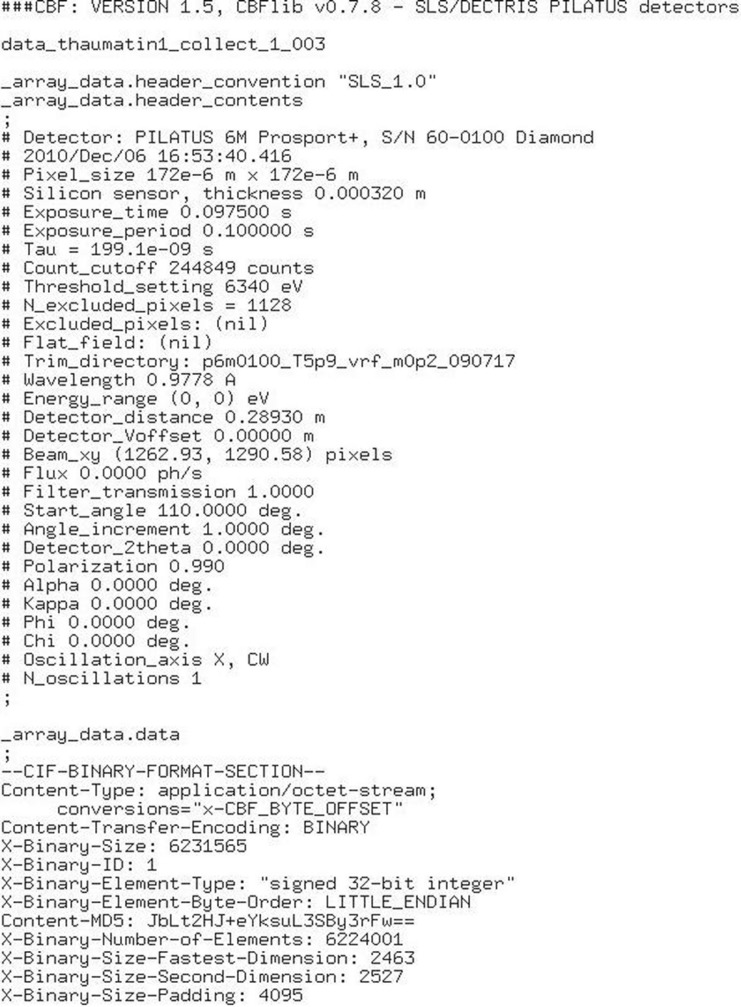
Mini-CBF header of the Dectris Pilatus detector.

An interesting tabulation of beamline settings for running *autoPROC* (Vonrhein *et al.*, 2011 ▸) is given at the website http://www.globalphasing.com/autoproc/wiki. Values such as BeamCentreFrom = header:x,-y, ReversePhi = ‘yes’ and TwoThetaAxis = ‘-1’ are given in order to cope with similar problems to those mentioned above (Table 2 ▸). There are eight possible ways in which the pixel values in the image file relate to the physical detector face, and detector vendors use all eight possible conventions (Wladek Minor, private communication). A wrong beam centre can hamper the indexing step. One can estimate the beam centre by manual inspection, by calibration using powder diffraction, by taking a direct beam shot or by removing Bragg spots and using the solvent diffuse ring to find the beam centre (Vonrhein *et al.*, 2011 ▸); otherwise one has to resort to trial and error. Fig. 7 ▸ shows the mini-CBF header that is used by Dectris for Pilatus detectors. Most of the information is present but some parameters are ambiguous: Beam_xy: see discussion above; Oscillation_axis is given as ’X’: what is the *X* direction? Polarization is 0.990: which plane has the strong intensity? We encountered an especially confusing situation where a Bruker fixed-χ goniometer was mounted with 90° rotation on Argonne beamline 15ID-B, while the images were converted to the normal Bruker instrument orientation. The strong polarization direction therefore appeared to be along the oscillation axis, but it was not (Jozef Kožíšek, private communication); only the string TARGET SYNCHROTRON in the header warned us.

More *a priori* knowledge is often needed to interpret diffraction image data. For example, there are different conventions on how to record dead regions on the detector: strips between detector panels on Pilatus detectors are indicated by ‘-1’, whereas in ADSC detector image files these are indicated by ‘0’. Data processing software has to interpret such pixel data correctly. Dark image and non-uniformity corrections may lead to negative intensities and some detector read-out handlers use a so-called baseline offset: a fixed integer number has been added to all pixel intensities to avoid having to store negative numbers. Removing the baseline offset is important in estimating the standard deviations of net Bragg reflection intensities and for measuring diffuse intensities between the Bragg peaks. Spatial distortion corrections are usually carried out and cannot be undone or corrected by processing software, but they affect standard deviations (Waterman & Evans, 2010 ▸) and this information should be conveyed in the metadata.

Detector hardware is being developed for high-speed serial crystallography experiments at X-ray free-electron laser (XFEL) installations or high-flux synchrotron beamlines that require ultra-fast data acquisition. A container HDF5 format, often with a NeXus data format layer on top, is designed for flexible and efficient input/output (I/O) for such high volumes of data. New data processing software packages such as *CrystFEL* (White *et al.*, 2012 ▸), *cctbx.xfel* (Sauter *et al.*, 2013 ▸) and *DIALS* (Waterman *et al.*, 2013 ▸) for serial crystallography are under development and this provides the opportunity to address the metadata issues anew.

**Figure 8:**
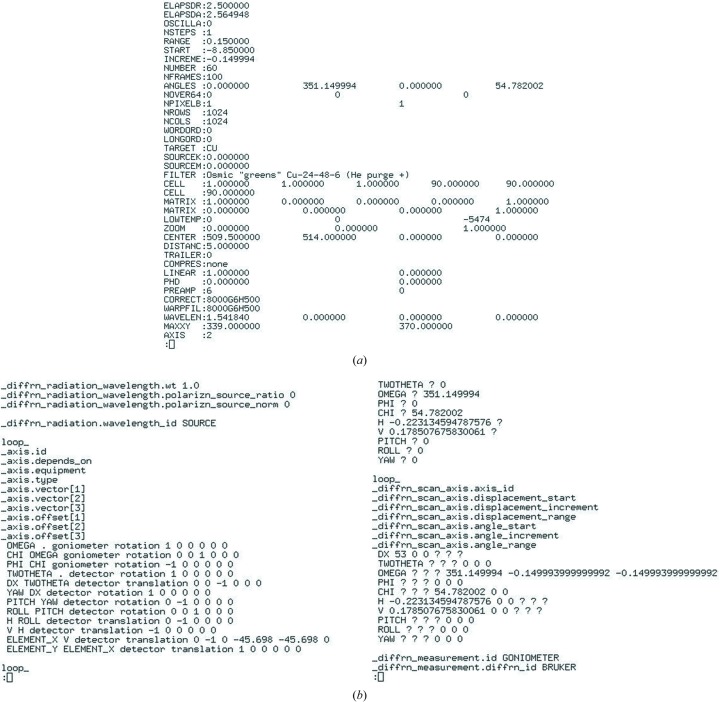
Comparison of header data in Bruker (*a*) .sfrm and (*b*) CBF formats.

Dectris has installed the Eiger detector at several synchrotron beamlines. Metadata are contained in a separate file (master.h5) linking to the image data files. The NeXus data representation (Könnecke *et al.*, 2015 ▸), like CIF, is very flexible and all metadata required can be captured by defining NeXus groups, fields and attributes. A good example of how consistent and comprehensive metadata can be stored in an imgCIF/CBF file is provided in Fig. 8 ▸ (Jörg Kaercher, Bruker AXS, private communication). In the proprietary Bruker .sfrm format the starting angles 2θ, ω, φ and χ are given (‘ANGLES: ...’). Their axis directions are not defined, whereas they are in the CBF format: the orientations and dependencies are given in the left-hand panel of Fig. 8 ▸(*b*). In .sfrm the rotation axis ‘AXIS: 2’ indicates ω, and the starting angle and increment are found at ‘START:’ and ‘INCREME:’; equivalent values are found in the CBF header at ‘_diffrn_scan_axis.displacement_angle’ and ‘_diffrn_scan_axis.displacement_increment’ (Fig. 8 ▸ *b*, right-hand panel).

## 4. A concern and an action arising from the Rovinj Diffraction Data Deposition Workshop   

A concern was voiced during open discussion at the workshop *via* the question ‘*Can we move away from the knowledge base in the various software packages, and make use of well developed metadata formats such as in CIF or NeXus?*’, *i.e.* a standardized raw diffraction image data format would make life easier for software developers but would require coordination between detector manufacturers. This has led directly to renewed calls for a standardized image format of appeal across the whole community. In conjunction with this question, the DDDWG is working on defining minimum requirements for metadata. We acknowledge that there will continue to be a great diversity of image formats (not least because of the existing installed base of detectors and the legacy data sets that have been archived), and conversion utilities such as *eiger2cbf* (https://github.com/biochem-fan/eiger2cbf) will continue to be needed. Nevertheless, it is important that anyone seeking to develop further new formats should be acutely aware of the need for adequate metadata characterization and interoperability that we have described above, and such an awareness may temper the proliferation of more new formats without particular demonstrable value.

In a separate discussion it was agreed that there is a need for a set of criteria for capturing and validating the essential experimental metadata for reproducibility of scientific results from any given raw data set. The proposal referred to this as ‘*checkCIF* for raw data’ and a close collaboration on this matter has been established with the IUCr COMCIFS (chaired by James Hester, who also attended the Rovinj Workshop). To develop these ideas further, a workshop run by the DDDWG is to take place at the ACA 2017 Conference in New Orleans in May 2017.

## 5. Concluding remarks   

In this topical review we have provided descriptions of the rapidly developing interest in and storage options for the preservation and reuse of raw data within the scientific domain supervised by the IUCr and its Commissions. We have highlighted the initiatives of science policy makers towards an ‘Open Science’ model within which crystallographers will work in the future; this will bring new funding opportunities but also new codes of procedure within open science frameworks. Skills education and training for crystallographers and frank discussion will be needed. Overall, we now have the means and the organization for preservation of our raw data, but still the need for careful thought about the metadata descriptors for each of the IUCr Commissions continues to be pressing. We note that the Commissions work within a diversity of instrumentation, and so a range of actions is required to improve on this current situation.

We have identified specifically the need to revisit the imperative for the community to adopt a standardized image format, and to agree at least a minimal set of essential metadata for reproducibility. The imgCIF dictionary (Hammersley *et al.*, 2005 ▸) is the natural starting point for the former, and the interaction between COMCIFS and NIAC (Könnecke *et al.*, 2015 ▸) demonstrates the feasibility of applying a common ontology across differing physical formats. There are also grounds for optimism that the idea of ‘*checkCIF* for raw data’ will appeal to both researchers and instrument vendors, given the enthusiastic representation of both at the Rovinj Workshop. As with all such initiatives, the rate of uptake will depend on drivers within the community. In the case of the original ‘*checkCIF*’ for derived data, structural science journals (especially those of the IUCr) that demanded relevant metadata and consistency checking provided one such important driver. In the case of raw data, which underpins all subsequent scientific deductions and derivations, we are encouraged by the emerging policies on research data management that we have summarized in this article, and by the many archiving initiatives that have sprung up around X-ray diffraction images in the space of the past few years.

## Figures and Tables

**Table 1:** A thematic raw data collection as an example: the suite of research studies, relating to platins binding to histidine, held at the University of Manchester Data Library

Entry No.	Raw diffraction data DOI	PDB code	Article DOI
1	10.15127/1.215887	4g4a (now 5hll)	10.1107/S1744309112042005 and 10.1107/S2053230X16000856
2	10.15127/1.219240	4dd2	10.1107/S0021889812044172 and 10.1107/S0907444912006907
3	10.15127/1.219241	4dd3	10.1107/S0021889812044172 and 10.1107/S0907444912006907
4	10.15127/1.219257	4dd9	10.1107/S0021889812044172 and 10.1107/S0907444912006907
5	10.15127/1.219267	4g4h	10.1107/S1744309112042005
6	10.15127/1.219263	4g4c	10.1107/S1744309112042005
7	10.15127/1.219242	4dd7	10.1107/S0021889812044172 and 10.1107/S0907444912006907
8	10.15127/1.219318	4gcb	10.1107/S090744491204423X
9	10.15127/1.219319	4gcc	10.1107/S090744491204423X
10	10.15127/1.219320	4gcd	10.1107/S090744491204423X
11	10.15127/1.219321	4gce	10.1107/S090744491204423X
12	10.15127/1.219322	4gcf	10.1107/S090744491204423X
13	10.15127/1.219260	4ddc	10.1107/S0021889812044172 and 10.1107/S0907444912006907
14	10.15127/1.219238	4ddb	10.1107/S0021889812044172 and 10.1107/S0907444912006907
15	10.15127/1.219230	4dd0	10.1107/S0021889812044172 and 10.1107/S0907444912006907
16	10.15127/1.219233	4dd4 (now 5l3h)	10.1107/S0021889812044172, 10.1107/S0907444912006907 and arXiv:1606.01372
17	10.15127/1.219236	4dd6 (now 5l3i)	10.1107/S0021889812044172, 10.1107/S0907444912006907 and arXiv:1606.01372
18	10.15127/1.219264	4g4b	10.1107/S1744309112042005
19	10.15127/1.219259	4dda	10.1107/S0021889812044172 and 10.1107/S0907444912006907
20	10.15127/1.219266	4g49	10.1107/S1744309112042005
21	10.15127/1.215883	4dd1	10.1107/S0021889812044172 and 10.1107/S0907444912006907
22	10.15127/1.266911	4nsj	10.1107/s2053230x14016161
23	10.15127/1.266910	4nsi	10.1107/s2053230x14016161
24	10.15127/1.266909	4nsh	10.1107/s2053230x14016161
25	10.15127/1.266908	4lt3	10.1107/s2053230x14016161
26	10.15127/1.266907	4lt0	10.1107/s2053230x14016161
27	10.15127/1.266906	4nsf (then 4xan now 5hmj)	10.1107/s2053230x14016161 and 10.1107/S2053230X16000777
28	10.15127/1.266905	4owb	10.1107/s2053230x14013995
29	10.15127/1.266904	4owa	10.1107/s2053230x14013995
30	10.15127/1.266903	4ow9	10.1107/s2053230x14013995
31	10.15127/1.266899	4mwk (now 5hmv)	10.1063/1.4883975 and 10.1063/1.4948613
32	10.15127/1.266900	4mwm (now 5hq1)	10.1063/1.4883975 and 10.1063/1.4948613
33	10.15127/1.266901	4mwn (now 5i5q)	10.1063/1.4883975 and 10.1063/1.4948613
34	10.15127/1.266902	4oxe (now 5idd)	10.1063/1.4883975 and 10.1063/1.4948613

**Table 2:** Implementation of goniometer types in *EVAL* (Schreurs *et al.*, 2010 ▸)

Goniometer	Axes, directions, off-set
Kappa	Axes: omega = *z*, kappa = *k*, phi = *z*, swing = *z*
	Rotation direction −1 −1 −1 −1
	Values: omega, kappa, phi, swing, dist
	Kappa support angle
Euler	Axes: omega = *z*, chi = *x*, phi = *z*, swing = *z*
	Rotation direction 1 1 1 1
	Values: omega, chi, phi, swing, dist
Horax	Axes: omega = *y*, chi = *x*, phi = *z*, swing = *y*
	Rotation direction 1 1 1 1
	Values: omega, chi, phi, swing, dist
DTB	Axes: omega = *z*, chi = −*x*, phi = *z*, swing = *y*
	Rotation direction −1 −1 −1 1
	Values: omega, chi, phi, swing, dist
X8	Axes: omega = *z*, chi = *x*, phi = *z* swing = *z*
	Rotation direction 1 −1 −1 1
	Values: omega+180, chi, phi+90, swing
X8C	Axes: omega = *z*, chi = *x*, phi = *z*, swing = *z*
	Rotation direction 1 −1 −1 1
	Values: omega+180, chi, phi+90, swing
Raxis	Axes: omega = *z*, chi = *x*, phi = *z*, swing = *z*
	Rotation direction −1 1 −1 1
	Values: omega, chi, phi, swing
Kappa180	Axes: omega = *z*, kappa = *k*, phi = *z*, swing = *z*
	Rotation direction: −1 −1 −1 −1
	Values: omega+180, kappa, phi, swing
	Kappa support angle
